# Sanitary Police Measures

**Published:** 1896-11

**Authors:** 


### SANITARY POLICE MEASURES MUST BE ADOPTED.

The cable brings news of an outbreak, among the people of Berlin, of a pustular character involving the mouth and throat, with severe inflammatory symptoms and great thirst. Prof. Virchow, after an examination, decided it to be due to infected milk, and says it simulates closely the foot-and-mouth disease. The dangers of consuming raw milk are gradually being better appreciated and are tending strongly toward the adoption of measures looking to greater safeguards and sanitary police regulations at the sources of production, thereby causing a closer surveillance of the animals producing this all-important food-product which has gone so long unchallenged as a disease-producer and conveyor. Measures of this kind must result in a better condition of things for all concerned, for they will add greater revenue to our agricultural population in the increased value of pure milk and its products; reduce the burdens of consumers by lessening disease among them, and decrease taxation for the support of hospitals, asylums, homes, and retreats that are not evidences of advancement when the cause of the evil that necessitates them goes unchallenged and undisturbed.

